# Score of fear of COVID-19 and physical activity level are related to the habitual consumption of dietary supplements

**DOI:** 10.1371/journal.pone.0307870

**Published:** 2024-09-06

**Authors:** Welligron Tavares da Silva, Paula Midori Castelo, Luciano José Pereira, Vanessa Pardi, Ramiro Mendonça Murata, Eric Francelino Andrade, Aline Carvalho Pereira

**Affiliations:** 1 Health Sciences Department, Universidade Federal de Lavras (UFLA), Lavras, Brazil; 2 Department of Pharmaceutical Sciences, Universidade Federal de São Paulo (UNIFESP), Diadema, Brazil; 3 Department of Foundational Sciences, School of Dental Medicine, East Carolina University (ECU), Greenville, North Carolina, United States of America; Universidade Federal de Minas Gerais, BRAZIL

## Abstract

The measures implemented to contain the COVID-19 pandemic resulted in both behavioral and lifestyle changes. The “Changes in Lifestyle-Related Behavior” instrument was developed to assess lifestyle-related behaviors in the Indian population. However, considering current knowledge, this instrument was not adapted for the Brazilian population. In addition, the relationship between fear of COVID-19 and consumption of dietary supplements has not yet been evaluated. Thus, we aimed to investigate the relationship between the use of dietary supplements with lifestyle behavior and the fear of COVID-19, as well as assess the psychometric properties of the Brazilian version of the “Changes in Lifestyle-Related Behavior” instrument. An online questionnaire assessed sociodemographic, occupational, anthropometric, physical activity (International Physical Activity Questionnaire—short form), fear of COVID-19, and lifestyle behavior data from 416 Brazilian adults (237 females; 18-60y). Mann-Whitney, Chi-square test, exploratory, and confirmatory analyses were applied. Exploratory and confirmatory analyses showed a satisfactory adequacy level of the questionnaire (CMIN/DF = 2.689; Cronbach’s α = 0.60) with 5 domains (‘Bad eating behavior’; ‘Healthy eating’; ‘Sleep quality’; ‘Interest in cooking’; ‘Number of portions and meals’). Lower fear of COVID-19 scores and higher levels of physical activity were found in participants who reported previous dietary supplement intake during the pandemic; in addition, the group that did not ingest dietary supplements reported greater changes in stress and anxiety levels during the pandemic (p<0.05). The intake of dietary supplements before the pandemic was associated with greater energy expenditure and better coping with the fear of COVID-19 during the pandemic. Additionally, the Changes in Lifestyle-Related Behavior tool can be used to assess lifestyle-related variables during the pandemic.

## Introduction

The outbreak of SARS-Cov-2 Coronavirus disease (COVID-19) initiated in Wuhan, province of China, has been declared a global pandemic on March 11, 2020 [1, 2]. To contain the spread of the disease, governments and main health agencies recommended the adoption of measures such as hand hygiene, mask use, and social isolation [3]. Such measures resulted in changes in lifestyle behavior, and the habits most affected during the pandemic were physical activity and eating behavior [4, 5].

In this sense, recent studies have proposed tools to assess aspects related to eating behavior and physical activity during the pandemic, in order to verify possible associations with other variables and to propose strategies to improve the population’s quality of life [6, 7]. Thus, Kumari et al. [8] developed an instrument consisting of 20 questions to assess changes in lifestyle-related behavior (diet, physical activity, and sleep) during the COVID-19 pandemic. This questionnaire showed satisfactory validity and good internal consistency, being considered well applicable to the Indian population as well as other Southeast Asian countries [8].

Considering eating behavior, the use of dietary supplements is an interesting variable to be investigated in the context of the pandemic, since this practice can influence other lifestyle behaviors [9]. The consumption of food supplements is associated with improvement in sports performance [10], immunity [11], and, even in psychological aspects [12, 13].

Considering that the pandemic exacerbated some psychological disorders such as anxiety and depression [14], it is important to investigate the relationship between the intake of dietary supplements and these disorders. The literature demonstrates that the increase in levels of anxiety and depression may be due to the fear of infection by COVID-19 [15, 16]. The fear of COVID-19 can influence lifestyle habits, as the individual changes their behavior in order to avoid situations with greater risk of contamination [17]. However, some behaviors may influence, positively, the fear of COVID-19 [18]. In this context, our primary objectives were to explore the relation between dietary supplement consumption and changes in lifestyle behavior, as well as to investigate the association with fear of COVID-19. Additionally, we aimed to translate the "Changes in Lifestyle-Related Behavior" questionnaire into Brazilian Portuguese and assess its psychometric properties.

## Methods

### Research design and sample characteristics

This cross-sectional study was performed on a sample of Brazilian individuals invited using the snowball online sampling method. The participants (Brazilian Portuguese speakers) filled out an online survey prepared on Google Forms (Alphabet, Mountain View, CA, USA) and sent via social networks (Facebook^®^, instagram^®^ and WhatsApp^®^). The sample comprised 461 participants (224 men and 237 women) aged between 18 and 60 years, residing in the Southeast region of Brazil. Based on statistical experience and the number of variables analyzed, a minimum of 400 observations was considered to conduct a multivariate analysis [19–21]. All participants were treated following the ethical guidelines of the Helsinki Declaration. The participants were provided with information about the aim of the study; they provided their written informed consent (online) to participate and were informed about the possibility of withdrawing from the study at any stage. The study was approved by the Research Ethics Committee of the Universidade Federal de Lavras (CAAE 51482921.7.0000.5148). All data were anonymized and are used in aggregate; no personal identifiers are reported.

We collected sociodemographic, occupational, and anthropometric data (age; gender; height; body mass; state/province; schooling; occupation; working from home or working from the office; tobacco habit; alcohol consumption; chronic diseases; COVID-19 diagnosis and COVID-19 complications). Participants also responded to the International Physical Activity Questionnaire (IPAQ—Short Form), the Fear of COVID-19 Scale (FCV-19S), and the Changes in Lifestyle-related Behaviour Questionnaire. Data collection occurred between September 27^th^ and October 29^th^, 2021.

### International Physical Activity Questionnaire (IPAQ—short form)

We assessed the participants’ intensity/frequency of physical activity through the modified version of the IPAQ-SF adapted to the COVID-19 pandemic context as described by Giustino et al., [22]. The original version of this self-reporting questionnaire allows to classify the level of physical activity performed in the last week according to patterns of walking, sitting, and exercise [23]. Due to the social isolation imposed by the COVID-19 pandemic, studies have adapted the IPAQ-SF to obtain information about the practice of physical activity before the quarantine, in order to compare the physical activity level between the two situations (before and during the pandemic of COVID-19) [22, 24]. Therefore, we converted the frequency and duration of physical activity data into a metabolic equivalent (MET), following the International IPAQ Committee guidelines [25, 26]. Thus, we classified each participant’s physical activity level according to the MET/min/week range (low level: <600 MET/min/week; moderate level: between 600 and 1499 MET/min/week; or high level: >1500 MET/min/week) [27, 28].

### Brazilian Portuguese version of Fear of COVID-19 Scale (BP-FCV-19S)

We used the Brazilian Portuguese version of the Fear of COVID-19 Scale (BP-FCV-19S) to assess the participant’s fear of COVID-19. The original scale was developed and validated by Ahorsu et al., [29] and properly validated for the Brazilian population by Andrade et al., [14]. This scale is composed by seven questions within a five-point Likert scale. During the validation of the BP-FCV-19S, the “physiological symptoms” and “emotional reactions” domains were identified. The score for each domain as well as the total score is calculated by the sum of the points. Thus, the greater the value, the greater the fear of COVID-19.

### Changes in Lifestyle-Related Behavior instrument

The Changes in Lifestyle-related Behavior is a self-reported questionnaire originally developed and validated for the Indian population [8]. This instrument allows to assess if there were changes in lifestyle-related behavior during the COVID pandemic. The questionnaire consists of 20 items covering the main information required to assess dietary habits (intake, meal pattern, and snack consumption), physical activity (duration and type), and sleep (duration and quality). During original validation, the authors identified six domains after the factor analysis that showed good internal consistency (Cronbach’s α = 0.72). However, the original study does not present enough information to classify the different domains.

In this study, we translated the items of the “*Changes in Lifestyle-related Behaviour*” into Brazilian Portuguese according to Andrade et al., (2021). Considering the semantic equivalence, a pre-test was carried out with the application of the translated version in 20 individuals (who were not included in the final study sample) to assess the acceptability and understanding of the questionnaire. These respondents were asked to complete the scale and, for each item, an additional “I didn’t understand” response was included; the researcher then checked whether or not they understood each question.

### Statistical analysis

Statistical analysis was performed using AMOS 25.0 and SPSS 27.0 software by an Applied Statistics specialist (PMC), considering an alpha level of 5%. Descriptive statistics consisted of means, standard deviation, medians, quartiles, and percentages. Normality was tested with the Kolmogorov-Smirnov test. To compare the groups of participants who consumed or not dietary supplements prior to the pandemic, continuous and categorical variables were compared using the Mann-Whitney and Chi-square tests, respectively.

The validity and reliability of the Changes in Lifestyle-Related Behavior questionnaire, translated into Brazilian Portuguese, were tested based on its psychometric properties. For exploratory and confirmatory factor analysis, information collected from 461 participants was randomly divided into two datasets: one for exploratory analysis (n = 230) and another for confirmatory analysis (n = 231) using Excel commands RANDOM () and ORDER EQ.

During exploratory analysis, the principal axis factoring was used to estimate the number of latent factors emerging from the questionnaire with 20 items. First, the correlation matrix was examined and the number of factors to be retained was based on the eigenvalues, the total explained variance, and the Scree plot. Since the factors could be correlated, the Oblimin rotation was used. The Kaiser-Meyer-Olkin general measure (KMO) and Bartlett’s sphericity test were examined as assumptions of the test.

The second data set was used for confirmatory factor analysis (n = 231). The model was built using the maximum likelihood method and considered the following assumptions: goodness of fit index (GFI), χ2 test, degrees of freedom, root mean square error of approximation (RMSEA), confirmatory fit (CFI), and standardized root mean square residuals (SRMR). The internal consistency of the overall score, as well as the score if items were excluded, were calculated using Cronbach’s alpha coefficient (Cronbach’s α), which measures the correlation between responses.

## Results

The sociodemographic and clinical characteristics of the respondents divided according to dietary supplement intake before the pandemic are shown in Table 1. Of the total number of respondents, 103 participants (22.3%) reported previous regular use of any of the following types of food supplement: Whey (64%), Creatine (46.6%), Vitamins (42.7%), Pre-Workout (22.3%), Amino Acids (8.7%), Hypercaloric (7.8%), Minerals (1.9%), Omega 3 (1.9), L-Carnitine (1%) and antioxidant (1%). Of these, 85.4% continued to consume dietary supplements during the pandemic, while of the participants who had not previously made regular use of supplements, 25% began to use some type of supplement during the pandemic.

**Table 1:** Participants’ characteristics regarding sociodemographic and clinical aspects, physical activity, and fear of COVID-19 divided according to the intake of dietary supplements before the pandemic (n = 461, data collection in 2021).

		Previous ingestion of food supplement (n = 103)	No previous ingestion of food supplement (n = 358)
**Age** [Mean (SD)]	Years	31.8 (8.2)	31.8 (9.0)
**Gender** (%men)		65†	44†
(%women)		35†	56†
**Education** (%)	Elementary School	3	3
	High school	19	27
	University education	45	40
	Postgraduate studies	33	30
**Occupation** (%)	Student	2	3
	Retired	1	0.5
	With occupation	97	95.5
	No occupation	0	1
**Remote work during pandemic** (%)		53	54
**Nutritional classification** (%)	Eutrophic	39†	45†
	Overweight	52†	34†
	Obesity	9†	21†
**Smoker** (%)		5	8
**Alcohol consumption** (%)		8	3
**Chronic disease** (%)		11	16
**Confirmed case of COVID-19** (%)		23	22
**Was hospitalized/complications from COVID-19** (n)		2	3
**Current use of food supplements** (%)		85	25
**METs LIGHT** [median (25–75%)]	Before the pandemic	808.5 (321.8–2400,8)	693.0 (198–1980)
**METs MODERATE** [median (25–75%)]		2160** (720–5760)	840** (0–2340)
**METs VIGOROUS** [median (25–75%)]		2400** (480–6660)	320** (0–2200)
**METs LIGHT** [median (25–75%)]	During the pandemic	792 (288,8–1980)	660 (99–1782)
**METs MODERATE** [median (25–75%)]		2160** (690–4800)	960** (0–2560)
**METs VIGOROUS** [median (25–75%)]		2280** (270–7680)	480** (0–4320)
**COVID-19 fear scale** [median (25–75%)]	Physiological symptoms	4* (3–6)	5* (3–6)
	Emotional reactions	11** (8–14)	13** (10–15)
	Total score	15** (13–19)	18** (14–21)

SD, standard deviation; METs, metabolic equivalent task minutes per week.

^†^ p<0.001, Chi-square test

* p<0.05, Mann-Whitney test

** p<0.001, Mann-Whitney test

The comparison of demographic data between groups that reported using or not any type of food supplement before the pandemic showed that a higher frequency of men and individuals classified as having normal weight (eutrophic) and a lower frequency of individuals with obesity comprised the group that used food supplement before the pandemic (p<0.001); there was no difference regarding age, schooling, occupation, frequency of tobacco and alcohol habits, chronic diseases and Sars-Cov2 infection (p>0.05).

Table 1 also shows the data regarding METs of physical activity performed and the fear of COVID-19 reported by participants. The analysis showed a difference in moderate and vigorous METS between the groups, both before and during the pandemic (p<0.001). The comparison of the total METS before the pandemic between the group that ingested (median = 6844.5) with that who did not ingest supplements before the pandemic (median = 3348), as well as the total METS during the pandemic (median = 6895.5 and 3810, respectively), also showed significant differences (p<0.001).

The comparison of the scores obtained in the Fear of COVID-19 scale between the groups also showed a significant difference in the two domains (physiological symptoms and emotional reactions) and in the total score (p<0.05), suggesting that the group that used food supplement before the pandemic, in addition to showing greater energy expenditure before and during the pandemic, also showed better coping of the fear of COVID-19.

Data regarding the reliability and validity of the Changes in Lifestyle-Related Behavior questionnaire and the results collected from participants’ responses regarding lifestyle changes during the pandemic are described below. In the pre-test carried out with 20 individuals to assess the acceptability and understanding of the scale, 100% of the individuals declared that they fully understood the instrument.

**Fig 1:**
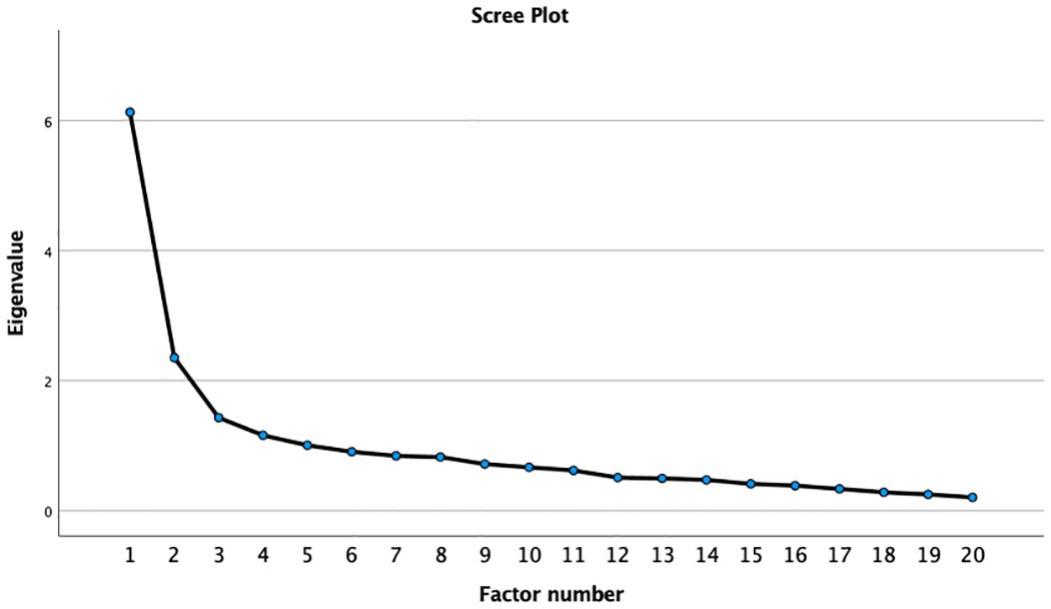
Scree plot used to observe the inflection point in the fifth factor.

Exploratory analysis was performed with data from 230 respondents; first, data normality was verified and, later, exploratory analysis was performed using principal axis factoring with Oblimin rotation. The KMO measure was 0.84 and the results of Bartlett’s sphericity test were X^2^ = 1719.16; DF = 190 and p <0.001, showing acceptable adjustment of the analysis; the Scree plot was used to observe the inflection point (Fig 1). Five factors were retained, which together explained 60.4% of the total variance of the data (Table 2). When six factors were retained (explaining 65% of the variance), the last domain consisted of only one question (Q1 “skipping meals”); therefore, the 5-factor solution (domains) was chosen.

**Table 2:** Factor matrix and regression coefficients: Factorization by the principal axis. Rotation method: Oblimin rotation (5 factors extracted; 9 iterations required). The highest loads are shown for each item (n = 461, data collection in 2021).

*Changes in Lifestyle-Related Behaviour*		Factor				
		1	2	3	4	5
Item	*Explained variance %*	*30*.*6*	*42*.*4*	*49*.*6*	*55*.*4*	*60*.*4*
1	*“skip meals”*	0.32				
2	*"snacking between meals"*					-0.41
3	*“portions of snacks and meals”*					-0.83
4	“*fruit and vegetable intake*”		0.77			
5	“*balanced diet intake*”		0.44			
6	“*intake of fast food and fried foods*”	0.67				
7	“*sugar-sweetened beverage intake*”	0.90				
8	“*ingestion of candies and sweets*”	0.76				
9	“*learn new recipes*”				-0.71	
10	“*consumption of unhealthy foods when stressed*”	0.55				
11	“*boost immunity food intake*”		0.87			
12	“*intake of food supplements that increase immunity*”		0.64			
13	“*support from family and friends for healthy eating*”		0.40			
14	“*learn healthy eating tips*”				-0.71	
15	“*participation in aerobic exercise*”		0.35			
16	“*participation in leisure and domestic activities*”		0.26			
17	“*time spent sitting*”	0.35				
18	“*changes in sleep hours*”			0.66		
19	“*sleep quality*”			0.73		
20	“*stress and anxiety level*”	0.38				

Factor 1: Bad eating behavior. Factor 2: Healthy eating. Factor 3: Sleep quality. Factor 4: Interest in cooking. Factor 5: Number of portions and meals.

Based on Table 2, it is possible to observe that five latent factors (domains) emerged: Factor 1 “Bad eating behavior” (questions 1, 6, 7, 8, 10, 17, and 20); Factor 2 “Healthy eating” (questions 4, 5, 11, 12, 13, 15 and 16); Factor 3 “Sleep quality” (questions 18 and 19); Factor 4 “Interest in cooking” (questions 9 and 14) and Factor 5 “Number of portions and meals” (questions 2 and 3).

**Fig 2:**
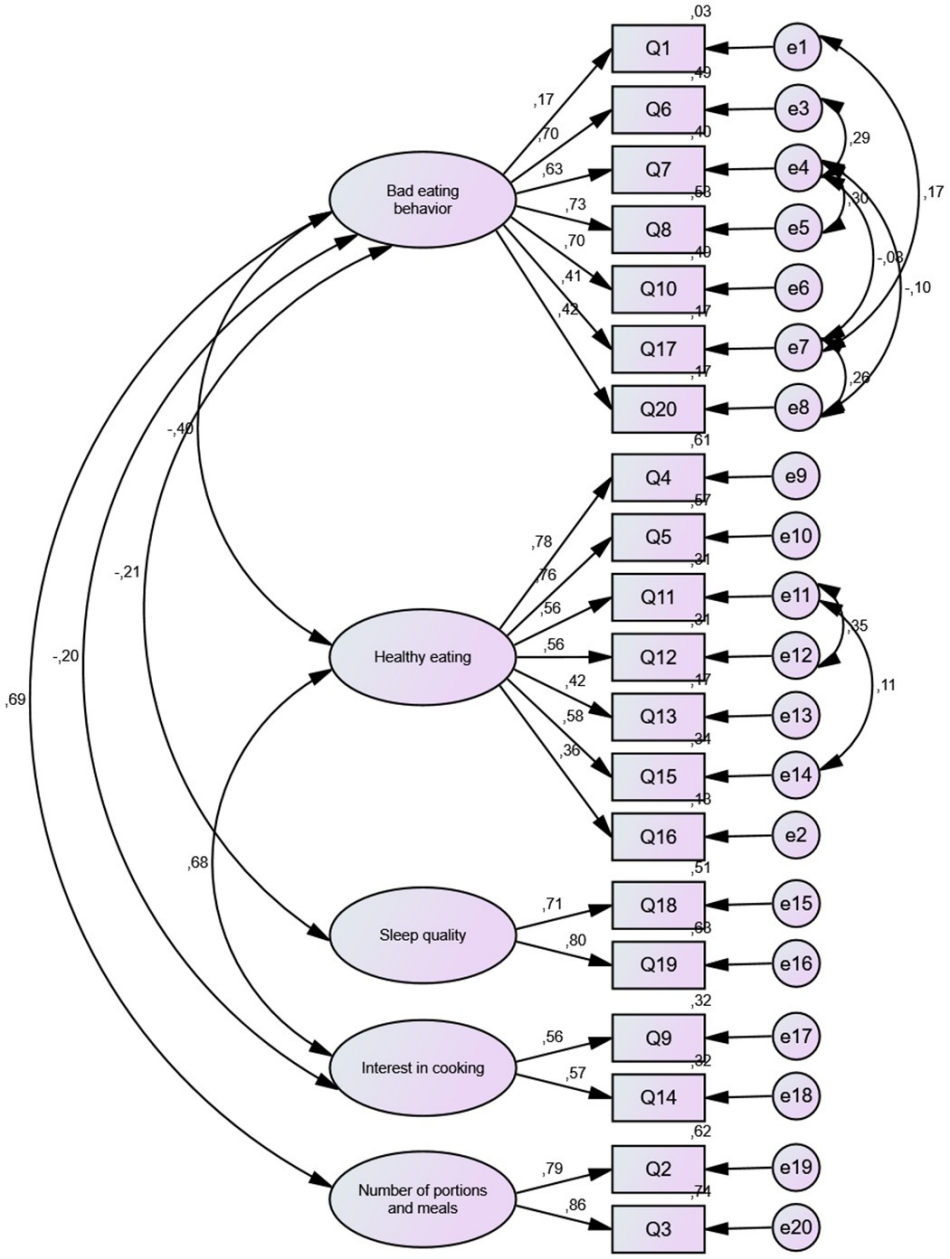
Path diagram defined *a priori* for confirmatory factor analysis and standardized regression coefficients of observed variables (Q1 to Q20) (p<0.05).

Confirmatory factor analysis was used to verify the adequacy level of the questionnaire with five domains, according to the model specified in Fig 2. The model showed a good fit according to the X^2^/DF index found (CMIN/DF = 2.689), although the other indexes have reached values below the ideal (GFI = 0.85; CFI = 0.83; RMSEA = 0.08 and SRMR = 0.087). The regression coefficients found for all questions and their respective domains were significant (p<0.01).

The questionnaire properties were also tested for reliability by obtaining the Cronbach’s α for the total scale and alpha coefficient if each item was excluded. Cronbach’s α obtained for the 20-item scale was 0.6; additionally, we observed that the coefficient did not increase substantially when each item was excluded (range between 0.55 and 0.61). Collectively, these results showed that the 20-item, 5-domain questionnaire has satisfactory psychometric properties [30].

The comparison of the scores obtained in the Changes in Lifestyle-Related Behavior questionnaire between the groups showed no significant difference in the scores of the five domains; the total score of the group that ingested any food supplement before the pandemic (median;25–75% = 65;61–68) and the group that did not ingest it (median;25–75% = 64;61–68) were also not different statistically (p = 0.607); only the score obtained in question 20 regarding the level of stress and anxiety differed between the groups, with the group that did not ingest a food supplement reported greater changes in levels of stress and anxiety during the pandemic (median; 25–75% = 4; 3–4) compared to the other group (median;25–75% = 3;3–4) (p = 0.037; Mann-Whitney test) (S1 Table).

## Discussion

Our main findings indicate that individuals who were using dietary supplements before the pandemic had lower fear of COVID-19 scores. Moreover, this group exhibited higher levels of physical activity (measured in METS) both before and during the pandemic. These results suggest that those who were using dietary supplements before the pandemic not only had increased energy expenditure during this period but also displayed better coping mechanisms in dealing with the fear of COVID-19. Furthermore, it is worth mentioning that this is the first study to carry out the confirmatory factor analysis of the Changes in Lifestyle-Related Behavior questionnaire, in addition to cultural adaptation and validation of the scale for the Brazilian population.

In our study, we investigated the consumption of dietary supplements, regardless of their purpose and we observed that the most consumed supplements were whey, creatine, and vitamins. Vitamin supplementation was associated with lower FCV-19S scores in women of reproductive age [31] while consumption of boosting immunity (vitamin and zinc supplements, honey, garlic, and medicinal plants) among Egyptians was associated with high fear of COVID-19 [32]. In a study carried out with a sample of Chinese participants, it was observed that the fear of COVID-19 was related to an enhanced purchase intention toward dietary supplements [33].

Furthermore, our findings indicate that individuals who regularly consumed dietary supplements had lower levels of fear related to COVID-19. It is possible that this behavior might serve as a coping strategy for the psychosocial effects of COVID-19 in this population. This relationship is concerning, as the use of dietary supplements should be approached with caution due to the potential for side effects, toxicity, and even psychological dependence on the use of certain compounds [34, 35]. Therefore, this association underscores the need for public policies that emphasize diets with the consumption of foods in their natural form, such as fruits and vegetables. Basic nutrition is capable of providing sufficient macronutrients and micronutrients for health maintenance without the need for nutrient-level reductionism [36, 37]. While the consumption of some types of supplements, such as vitamins, probiotics, and certain trace minerals, is associated with the improvement of the immune response [13, 38], in the sample evaluated, we did not observe an association between the consumption of dietary supplements and lower severity of complications resulting from COVID-19. During the COVID-19 pandemic, there was an increase in the dissemination of information, without scientific evidence, regarding miraculous supplements to boost immunity and combat infection [39]. This fact can also justify the increase in the consumption of dietary supplements during this period [9]. It is important to emphasize that the use of dietary supplements does not replace basic nutrition and healthy food consumption [40]. In fact, the incorrect use of these agents can be detrimental to health [41]. In this way, to avoid side effects and improper use of dietary supplements, the population needs to adhere to dietary recommendations promoted by health agencies, such as the Brazilian Food Guidelines [42, 43].

We observed that the use of supplements was associated with higher levels of physical activity. Thus, we speculate that the lower fear of COVID-19 scores in participants who use supplements may also be related to a higher level of physical activity. This relationship was observed in previous studies where the practice of physical activity was related to lower COVID-19 fear scores [44, 45]. Physical activity can modulate anxiety, which is one of the main components of fear [14, 46]. Moreover, physical activity promotes an immediate positive effect on mood and feelings of energy as well as leads to a distraction from negative thoughts and stress related to the COVID-19 infection [45]. Additionally, although associations were not observed between the use of supplements and most items in the "Changes in Lifestyle-Related Behavior" questionnaire, we found that individuals who consumed dietary supplements reported experiencing fewer changes in their stress and anxiety perception during the pandemic, as observed in question 20 of this instrument. The lower perception of these mental health outcomes in individuals who consume dietary supplements may be associated with the fact that, generally, these individuals exhibit greater self-care in terms of diet and overall health, which is reflected in anxiety and depression levels [47]. Additionally, the consumption of certain compounds may induce a placebo effect on mental health determinants [48]. In these cases, individuals may take dietary supplements believing that they will improve their anxiety and depression levels, and this improvement may occur even without a physiological mechanism to justify the outcome [49].

An interesting result observed in this sample was the higher percentage of participants classified as overweight who consumed dietary supplements before the pandemic. A closer look at this result showed that these participants performed physical activity at moderate and high levels. Thus, it is possible to infer that this classification as overweight may be related to a high amount of muscle mass, and not necessarily, body fat.

Considering the psychometric properties of the Changes in Lifestyle-Related Behavior questionnaire, the instrument showed satisfactory results, with a Cronbach’ α equal to 0.60, while the original scale showed a Cronbach’ α equal to 0.72 [8]. In addition, the exploratory and confirmatory analyses identified five domains (“bad eating behavior”, “health eating”, “sleep quality”, “interest in cooking”, and “number of portions and meals”), instead of the six domains suggested in the original study. No further detail was found in the study regarding the confirmatory analysis [8], although reaching satisfactory results, data collectively point to the need for changes in the original questionnaire in order to increase internal consistency. Future studies should make adjustments to the number of questions per domain and readjust the inter-relatedness between items, which are factors that influence the alpha value [30].

Our findings collectively reinforce the study’s robustness and its valuable contribution to the existing literature on the relationship between dietary supplement use, lifestyle behaviors, and psychological outcomes during the COVID-19 pandemic. At our knowledge, this is the first study to assess the relationship between these variables in the same study design.

## Conclusions

Our analysis revealed that males with higher energy expenditure in moderate and intense activities, and a lower perception of fear of COVID-19 tended to use dietary supplements. Furthermore, individuals who consumed supplements reported experiencing fewer changes in stress and anxiety levels during the pandemic. These findings suggest that supplement users not only had increased energy expenditure but also demonstrated better coping mechanisms in dealing with the fear of COVID-19. Additionally, our study contributes by being the first to conduct a confirmatory factor analysis of the Changes in Lifestyle-Related Behavior questionnaire, along with the cultural adaptation and validation of the scale for the Brazilian population. We believe that the increase in the consumption of dietary supplements observed during the pandemic in the present study was due to greater exposure to information without scientific evidence. Thus, our research highlights the importance of public policies that can educate people about the potential dangers of using dietary supplements without professional guidance.

## Supporting information

S1 Table Comparison of the changes in Lifestyle-Related Behavior questionnaire results between the groups of participants divided according to the intake of dietary supplements before the pandemic. Mean (SD) and [MED] are shown.
(DOCX)
