# Supplementary material for: Score of fear of COVID-19 and physical activity level are related to the habitual consumption of dietary supplements
Source: PLoS One. 2024 Sep 6;19(9):e0307870. doi: 10.1371/journal.pone.0307870 (PMC11379318; doi:10.1371/journal.pone.0307870)
Supplement: S1 Table — Mean (SD) and [MED] are shown. (DOCX) [file pone.0307870.s001.docx]

**S1 Table.** Comparison of the Changes in Lifestyle-Related Behavior Questionnaire results between the groups of participants divided according to the intake of dietary supplements before the pandemic. Mean (SD) and [MED] are shown

| **Question** | **Previous ingestion of food supplement**  **(n=103)** | **No previous ingestion of food supplement**  **(n=358)** |
| --- | --- | --- |
| Q1 | 3.0 (0.9) [3] | 3.2 (0.9) [3] |
| Q2 | 3.2 (0.9) [3] | 3.3 (0.9) [3] |
| Q3 | 3.3 (0.8) [3] | 3.3 (0.9) [3] |
| Q4 | 3.4 (0.9) [3] | 3.2 (0.9) [3] |
| Q5 | 3.3 (0.8) [3] | 3.2 (0.9) [3] |
| Q6 | 3.1 (1.0) [3] | 3.2 (1.0) [3] |
| Q7 | 2.8 (1.0) [3] | 3.0 (1.0) [3] |
| Q8 | 3.0 (1.0) [3] | 3.2 (0.9) [3] |
| Q9 | 3.4 (0.7) [3] | 3.3 (0.8) [3] |
| Q10 | 3.3 (0.9) [3] | 3.1 (0.9) [3] |
| Q11 | 3.4 (0.8) [3] | 3.4 (0.9) [3] |
| Q12 | 3.2 (0.9) [3] | 3.2 (0.8) [3] |
| Q13 | 3.3 (0.8) [3] | 3.3 (0.8) [3] |
| Q14 | 3.4 (0.8) [3] | 3.4 (0.8) [3] |
| Q15 | 3.4 (1.0) [3] | 3.2 (1.1) [3] |
| Q16 | 3.3 (0.8) [3] | 3.3 (0.9) [3] |
| Q17 | 3.6 (1.1) [3] | 3.6 (1.0) [3] |
| Q18 | 2.9 (0.6) [3] | 2.9 (0.8) [3] |
| Q19 | 2.9 (0.7) [3] | 2.8 (0.8) [3] |
| Q20 | 3.5 (0.9) [3]* | 3.7 (0.9) [4]* |
| **Total** | 64.9 (5.2) [65] | 64.8 (4.8) [64] |

SD, standard deviation; MED, median.

*p=0.037; Mann-Whitney test.
